# Supplementary material for: Sustaining indigenous Maasai Alalili silvo-pastoral conservation systems for improved community livelihood and biodiversity conservation in East African rangelands
Source: PLoS One. 2024 May 16;19(5):e0303649. doi: 10.1371/journal.pone.0303649 (PMC11098305; doi:10.1371/journal.pone.0303649)
Supplement: Attachment — Submitted filename: Response to Reviewers.docx [file pone.0303649.s003.docx]

The Nelson Mandela African Institution of Science and Technology,

P.O.BOX 447,

Arusha-Tanzania.

E-mail: [elkana.hezron@nm-aist.ac.tz](mailto:elkana.hezron@nm-aist.ac.tz)

Mobile No: +255 766 014 232

12^th^ February, 2024

Dr. Emily Chenette (Editor – in – Chief),

PLOS ONE - Public Library of Science.

Dear Sir/Madam,

**RE: Manuscript titled: Sustaining indigenous *Maasai* *Alalili* silvo-pastoral conservation systems for improved community livelihood and biodiversity conservation in East African rangelands**

Refer to the subject above.

Following the revision of our aforementioned article, hereby we would like to re-submit the manuscript for publication consideration in PLOS ONE.

This article is re-submitted to PLOS ONE after addressing all comments as were raised by the editor and reviewers (Table below). All authors have agreed to the re-submission of the manuscript to your journal, and have no conflict of interest. The funders had no role in study design, data collection and analysis, decision to publish, or preparation of the manuscript.

We will be delighted if you consider our manuscript acceptable for publication in PLOS ONE.

Sincerely on behalf of the authors

Elkana Hezron

**EDITOR’s COMMENTS ADDRESSED**

| **General comment** | | **Response** |
| --- | --- | --- |
| Thank you for submitting your manuscript to PLOS ONE. After careful consideration, we feel that it has merit but does not fully meet PLOS ONE’s publication criteria as it currently stands. Therefore, we invite you to submit a revised version of the manuscript that addresses the points raised during the review process. | | Thank you for the compliment, we have addressed all the comments that were raised by reviewers as indicated in this table and the revised manuscript. |
|  | | |
| **Other specific comments** | | |
| **S/N** | **Comments** | **Response** |
| 1. | Please ensure that your manuscript meets PLOS ONE's style requirements, including those for file naming. | We have considered the PLOS ONE’s style requirements in the revised manuscript |
| 2. | Thank you for submitting the above manuscript to PLOS ONE. During our internal evaluation of the manuscript, we found significant text overlap between your submission and previous work in the [introduction, conclusion, etc.]. | Thank you for the observation, the previous work was a project report that was  submitted to the Rufford Foundation. The funder has no conflict of interest, the report was a requirement for justifying the project outputs.  However, the text overlap has been worked out and addressed as recommended in the revised manuscript. |
| 3. | We note that the grant information you provided in the ‘Funding Information’ and ‘Financial Disclosure’ sections do not match.  When you resubmit, please ensure that you provide the correct grant numbers for the awards you received for your study in the ‘Funding Information’ section. | We have revised the grant numbers to ensure that they are matched in the “Funding Information” and “Financial Disclosure” sections |
| 4. | Thank you for stating the following financial disclosure:  "This work was funded by the Nelson Mandela African Institution of Science and Technology through African Development Bank (AfDB) (Grant No: P-Z1-IA0-016), Higher Education for Economic Transformation (HEET) project (Grant No: IDA68870) as well as the Rufford small research grants (Grant No: 37388-1). "  Please state what role the funders took in the study. If the funders had no role, please state: "The funders had no role in study design, data collection and analysis, decision to publish, or preparation of the manuscript."  If this statement is not correct you must amend it as needed.  Please include this amended Role of Funder statement in your cover letter; we will change the online submission form on your behalf. | Thank you for the observation, the statement has been included in the cover letter as suggested. |
| 5. | We note that your Data Availability Statement is currently as follows: [All relevant data are within the manuscript and its Supporting Information files.]  Please confirm at this time whether or not your submission contains all raw data required to replicate the results of your study. Authors must share the “minimal data set” for their submission. | We would like to confirm that our submission contains a minimal raw data set required to replicate the results of this study. |
| 6. | Your ethics statement should only appear in the Methods section of your manuscript. If your ethics statement is written in any section besides the Methods, please delete it from any other section. | The ethics statement has been written in the Methods section only (section 2.3) as suggested |
| 7. | We note that [Figures 1, 6, 7 and 8] in your submission contain [map/satellite] images which may be copyrighted. All PLOS content is published under the Creative Commons Attribution License (CC BY 4.0), which means that the manuscript, images, and Supporting Information files will be freely available online, and any third party is permitted to access, download, copy, distribute, and use these materials in any way, even commercially, with proper attribution. For these reasons, we cannot publish previously copyrighted maps or satellite images created using proprietary data, such as Google software (Google Maps, Street View, and Earth). For more information, see our copyright guidelines: http://journals.plos.org/plosone/s/licenses-and-copyright.    We require you to either (1) present written permission from the copyright holder to publish these figures specifically under the CC BY 4.0 license, or (2) remove the figures from your submission: | Thank you for observation, some contents in this manuscript are the extract of our recently submitted Rufford project report. We have submitted a written permission from the funder (Rufford) signed by copyright holder (corresponding author) to publish the mentioned figures under the CC BY 4.0 license as suggested. |

**REVIEWER #1: COMMENTS ADDRESSED**

| **General comment** | | **Response** | |
| --- | --- | --- | --- |
| The research paper employs a cross-sectional research design to investigate the existing Maasai Alalili silvo-pastoral conservation systems in East African rangelands. The findings reveal that while these systems are still present, their sustainability is under significant threat.  Here are some suggestions for enhancing the clarity and robustness of the paper: | | Thank you | |
|  | | | |
| **Other specific comments** | | | |
| **S/N** | **Comments** | **Response** | **Page/line No.** |
| 1. | Address the lack of supporting evidence for the claim about decreasing Alalili sizes by including graphics, analyses, or references. | The supporting evidence for the decreasing Alalili sizes has been added as suggested | Page #6  Lines 96, 99 |
| 2. | Consider exploring the relationship between Alalili size and livestock herd size. | The association between Alalili size and the livestock herd size grazed in the Alalili have been added as suggested | Page #7  Line 112  Page #12  Lines 216  Page #15  Line 282  Page #25  Lines 488 – 500  Page #28  Line 560 |
| 3. | Additionally, expand the literature review to encompass a similar system in southern Kenya known as "Olopololi" (refer to <https://amboseliecosystem.org/our-campaigns/#:~:text=01.-,Olopololi%20Plots,-The%20campaign%20is>). | The literature review has been expanded as suggested to include the Olopololi | Page #4, 5  Lines 56 – 58, 63 |
| 4. | Provide exact p-values for all values greater than or equal to 0.001, adhering to PLOS ONE Submission guidelines. | The p-values for all values greater than or equal to 0.001 have been provided as suggested, and P-values less than 0.001 are expressed as p < 0.001 | Page #17  Lines 332, 338, 340  Page #18  Lines 353, 356  Page #19  Line 358  Page #21  Lines 411, 417  Page #24 – 25  Lines 472, 474, 483, 485, 487 |
| 5. | Enhance figure captions by incorporating additional text to clarify the figure contents. | Additional text has been added to the figure captions to clarify the figure content as suggested | Page #8  Line 138  Page #17  Line 335  Page #19  Line 362  Page #21  Line 408  Page #22  Line 426 |
| 6. | Cite QGIS and other software/tools used in the analysis. | All software/tools used for analysis have been cited as suggested | Page #11  Line 195  Page #14  Line 266  Page #15  Line 283 |
| 7. | Improve the clarity of the formulation and interpretation of the Generalized Estimation Equation (GEE) model in Table 3. Clearly define the response and explanatory variables, specify categories used as references, and offer detailed model information in the supplementary material to assist readers in understanding the analysis. | The GEE model equations have been added as suggested and the variables have also been defined in section 2.6 while specifying the response and explanatory variables as well as categories that were used as reference variables. We did not include the descriptions along with tables 3 and 4 to avoid repetition of the same information that already appears in the methodology section 2.6 | Page #15  Lines 284 – 290 |

**REVIEWER #2: COMMENTS ADDRESSED**

| **General comments** | | **Response** | **Page/line No.** |
| --- | --- | --- | --- |
| 1. | Although the indigenous Maasai Alalili silvo-pastoral conservation systems have been successful in the past, it is no longer sustainable given the current human population growth, allocation of lands to conservation, climatic and socio-political tendencies in northern Tanzania. In addition, droughts have decimated rangelands, and many pastoral communities in Ngorongoro district are being relocated to other regions of the country as part of the government land use transformation plans. Despite the importance of the Maasai Alalili silvo-pastoral conservation systems, current government policies would suggest that the practice might phase out in the next few years. The impacts are already obvious. Hence, I would expect the authors to integrate these issues in relevant parts of the manuscript. For example, how would these current policies affect the Maasai Alalili silvo-pastoral conservation systems? | Thank you for the observation, these issues have been integrated in relevant parts of the manuscript as suggested | Page #6  Lines 81 – 94 |
| 2. | In the data collection process, insufficient information was provided on how the mapping process was conducted and how the authors could have determined the sizes of each rangeland/Alalili. These details are important to allow reproducibility of the results that determine the sizes of each Alalili, which are key results of the manuscript. | The mapping process as well as the procedure followed for size determination through measurement of the area covered by the surveyed Alalili have been added in the respective section as recommended. | Page #12 – 13  Lines 231 - 252 |
| 3. | Although using Google satellite images for northern Tanzania might be a reasonable verification process, these are time sensitive information, and the image dimensions and size of rangeland will depend on the season they were captured. In addition, Google satellite images for low resource settings are usually several years old. | The inquired details for Google satellite images to digitize the sampled Alalili systems have been described as recommended in the respective section | Page #11  Line 194  Page #12  Line 220, 231 – 233  Page #13  Lines 235 – 252 |
| 4. | The methods for classification of communal and private Alalili should be integrated as part of the study design. I would expect such important data to be collected through a standard process with detail methodology on how the participatory process was conducted, number of participants per village and category of individuals etc. Similar details would be required for the semi-structured interviews. Appending a statement at the end of the paragraph that participatory field visits and semi-structured interviews were used is not very convincing because these are methods that require substantial details. | We are grateful for this observation and we have provided an adequate description of the classification methods of the surveyed Alalili systems in section 2.5  The participatory field visits have been described and the modality through which semi-structured interviews were conducted has been added coupled with guiding questionnaires and checklists. | Page #12-13  Lines 254 – 255 |
| 5. | The discussion section was also not clear and difficult to follow: on one hand the authors were making a case for more communal Alalili systems given that they are better managed; and on the other hand there were suggestions that efforts should be directed towards increasing the number and size of private Alalili. | The discussion section has been revised to address your observation and the surveyed literature to make the comparison understandable | Page #27  Lines 523 – 541  Page #28  Line 544 – 562  Page #29  Line 582 |
|  | | | |
| **Specific major comments** | | | |
| **Abstract** | | | |
| **S/N** | **Comments** | **Response** | **Page/line No.** |
| 1. | A brief definition of the Alalili systems in the context of other systems should have been described in the beginning of the abstract. | Thank you for the observation, a brief definition of Alalili systems have been added in the introduction as suggested | Page #2  Line 10 |
| 2. | Line 18: What proportion of the communal systems have vanished? How many are left? Adding more quantitative and statistical information when findings are presented is important for the reader to understand the evidence. | The abstract section has been corrected by adding the relevant quantitative data and statistical description as suggested. | Page #2  Line 22 |
|  | | | |
| **Introduction** | | | |
| **S/N** | **Comments** | **Response** | **Page/line No.** |
| 3. | Line 68: In Tanzania what proportion of livestock are kept through Ngitili, Milanga is Radanenda and Alalili? | As of a recent literature survey, the proportion of livestock being kept under the traditional rangeland conservation systems has been not estimated. However, we managed to conduct an estimation survey for the current proportion of livestock accommodated by Alalili systems. Thus, it remains an existing research gap for further studies. | Page #7  Lines 112 – 114 |
| 4. | Does all Maasai population practice Alalili? If not, what are the other major types of surviving strategies the Maasai use? | Based on the literature and actual surveys conducted in the Maasai land, all Maasai population in northern Tanzania have been practicing Alalili although, they rely on the general grazing lands open to every household. However, not all villages or households possess adequate Alalili systems during times of pasture scarcity, thus they are reported to request grazing in the secured Alalili of the neighboring villages or households. This is the reason we are interested in mainstreaming this practice throughout the Maasai pastoral community which seems to be the appropriate solution to pasture scarcity. | Page #6  Line 81 |
|  | | | |
| **Methods** | | | |
| **S/N** | **Comments** | **Response** | **Page/line No.** |
| 5. | Line 90: What districts are the Maasai pastoral society located in in Northern Tanzania? | The districts in which the Maasai pastoral society of Northern Tanzania are located have been added in section 2.1 | Page #7  Lines 120, 121 |
| 6. | Line 162-163: What are the justifications for the sampling methods used, e.g. why was a combination of purposive and/or purposive stratified sampling techniques used in the study? | The study area where the field survey was conducted is categorized into more than one land use practice thus, we used stratified sampling. On the other hand, we aimed to study the Maasai Alalili systems, thus we employed purposive sampling of the districts where Alalili can be accessed while randomization was undertaken to enhance the unbiased sampling process as we have addressed in the respective section 2.4 | Page #11 – 12  Lines 199, 213, 219 |
| 7. | Line 167: These details fit better in the study area description. | Thank you for the observation, the details have been shifted to the description of the study area in section 2.1. as suggested | Page #7  Lines 120, 121 |
| 8. | Line 175- 179: How many key informant interviews were conducted per district to identify villages that practice Alalili systems? A village was the sampling unit? Where in the districts were these interviews conducted? Was the interview conducted just once for each district? This must be very clear because it an important aspect of the methods, given that the interviews provided valuable information that informed the list of all villages that practice Alalili systems? If so, how was members of the VEO/WEO, and members of village rangeland management committee selected? These individuals are unique for each village in the districts. For example, Longido has more than 51 villages and each village has a VEO. | The technique of conducting the key informant interviews and the respective number has been described as recommended in the section 2.4.  However, it was difficult to reach out every VEO in every village considering that some VEO administers more than one village at the same time.  Therefore, DGOs and ROs of the respective sample districts assisted to contact them through phone calls as well as coordinating communications between WEO/VEO and the leaders of the village rangeland management committees to execute the listing of all Alalili available in their villages. | Page #11  Lines 206, 209 |
| 9. | Line 179-180: Again, please clarify “A total of 298 Alalili systems…”. Are these the number of villages (that is the sampling unit) that practice Alalili systems out of all (n=?) the villages in study districts? What systems does these other villages or communities or clans practice? Why 40%, what are the justification for the sample size of 119? | We are grateful for this comment. The inquiries have been addressed in the respective section 2.4.  Also, we would like to clarify that the 298 represents a total count of all Alalili silvo-pastoral conservation systems that were listed from the key informants in their respective villages as per coordination made by DGOs and ROs of each district.  119 represents the 40% of 298 Alalili identified. We considered surveying the 40% of the Alalili systems to ensure broad coverage of the sampled study area which is featured by different land use categories to enhance its characterization process. Therefore, we aimed to capture the real reflection of Alalili abundance and its distribution status throughout the rangeland of northern Tanzania. We thought it would have not brought a sense of reality to have a representation in wide scope while having only a few sample Alalili systems.  On the other hand, our study was focused on characterizing this traditional practice and mainstreaming it into rangeland restoration initiatives and an enhanced livelihood sustainability. | Pages #11 – 12  Lines 211 – 217 |
| 10. | Line 182: How was the Empirical evidence-based method (Ground-truthing technique) performed? | An explanation on how ground truthing technique was done has been added. | Page #12  Line 227 |
| 11. | Lines 185-196: More detail is required throughout this section. Every single village sampled was visited and mapped to verify the exact land use types? So, how was the ground-truthing data collected in each village? For example, as indicated in S1 table, a single waypoint/coordinates for each Alalili system/rangeland? What location was the waypoint collected, centre or edge of the Alalili? Four radii were recorded from the centre to the north, south, east and west directions assuming that Alalili has irregular polygon- how was this recorded (e.g. using transect), and with what instruments (e.g. handheld GPS and paper maps)? And where are the results, given that the local field assistant for each specific village only recorded the average radius, and only a single waypoint was recorded in the S1 table? These details are important to allow reproducibility of the results that determine the sizes of each Alalili, which are important results for the manuscript. Line 191: How was the verification performed? What was the year and date the Google satellite image in QGIS environment was captured? How was the digitisation process performed? | The suggestions have been considered and the required details have been added in the manuscript at section 2.5.  The study aimed at surveying and characterizing the sampled Alalili systems in terms of the currently existing types, sizes, abundance, and distribution that are so far lacking in the modern world kinds of literature and scientific records needed for enhanced rangeland management and conservation strategies.  Therefore, each sample Alalili in the respective villages, where they were initially identified by the key informants, was visited and the DGOs/ROs helped to determine the actual land use where the Alalili was found. The member of the village rangeland management committee (local field assistants) helped to identify the type of the visited Alalili systems, i.e., communal or private. This is because the types were not known following the random sampling technique used to obtain the sample Alalili sites for survey.  A single GPS coordinate was recorded from the center of each surveyed Alalili to enhance mapping of their distribution and abundance as portrayed in the respective figures.  The digitization of each Alalili was done using Google Earth Pro with the image captured by Landsat/Copernicus at a date ranging between February 2018 to December 2020. The local field assistant helped to identify the boundaries of each Alalili in the zoomed satellite view on the screen. | Page #12 – 13  Lines 232 – 252 |
| 12. | Lines 195: What are participatory visits? How were the semi-structured interviews conducted? Where is the questionnaire? | The participatory field visits have been described and the modality through which semi-structured interviews were conducted has been added coupled with guiding questionnaires and checklists. | Pages #13 – 14  Lines 255 – 258 |
|  | | | |
| **Results** | | | |
| **S/N** | **Comments** | **Response** | **Page/line No.** |
| 13. | Line 222: Again, it is difficult to understand this classification when the method description was not adequate. | We are grateful for this observation and we have provided an adequate description of the classification methods of the surveyed Alalili systems in section 2.5 | Page #14  Lines 260 – 262  Page #15  Line 297 |
| 14. | Figure 5: Are there no other types of land uses in the study area? | The land uses mapped in this study considered the land use plan in the northern Tanzania regions described by the Land Act of Tanzania Cap. 113 of 1999, Local Government (District Authorities) Act Cap. 287 and the Land Tenure (Village Settlements) Act No. 27 of 1965, the Wildlife Conservation Act of 1974[Cap. 283] and wildlife policy endorsed in 1988. (Described in section 2.2). | Attached Figure 5 |
| 15. | Figures 6 and 7: What information was used for this map classification? Ngorongoro district has NCA, Loliondo Game Controlled Area and village lands with significant human settlements around Loliondo town and Wasso based on 2012 census. Has this changed? Longido also has village lands around Longido town and Namanga wards. | The stratification of land use in the maps was done based on the shape files collected from Tanzania Wildlife Research Institute (TAWIRI).  However, we agree that Ngorongoro district has NCA, Loliondo GCA, and village lands with reference made to the prior information given in the above government land policies, but Loliondo was not included in the proposed area for survey due to the emerged conflicts of interest about land tenure. Therefore, we assumed that Alalili in the Loliondo GCA and its adjacent village lands would be well represented by the surveyed rangeland areas. | Attached Figures 6 and 7 |
|  | | | |
| **Discussion** | | | |
| 16. | Line 426 - 438: This discussion is not very clear because Maasai rangelands in northern Tanzania are largely communal with a small proportion being private, which is expected given that Maasai communities in Tanzania have not partition communal lands as it is currently being done in Kenya. Therefore, the result does not suggest that household level practice is being threatened. In fact, it is the opposite as more communal lands (18%) are being acquired by individual for raising livestock and other personal use. The result present does not show if this situation has changed. | Thank you for observation, the discussion section has been revised to match the results and the existing literary works. | Page #27  Lines 526 – 541  Page #28  Line 544 – 550  Page #29  Line 582 |
| 17. | Line 428: Again, the results do not show any changes, it only categorises rangeland across land use types. | We are grateful for the comment and wish to accept its strength in improving our manuscript.  Being conducted for the first time, the results section presents the variation in Alalili abundance and distribution across identified types, size categories, and surveyed land use categories. The discussion section describes the status of Alalili and the significance of its existence in sustaining the rangelands of eastern Africa which are faced with various pressures. Therefore, we are suggesting the Maasai Alalili practices to be used as an important indigenous strategy that can be easily adapted by Maasai pastoralists to restore the degraded rangelands while conserving the ecosystems for sustainable development and livelihood improvement. | Page #30  Line 596 |
|  | | | |
| **Minor comments:** | | | |
| **Introduction** | | | |
| **S/N** | **Comments** | **Response** | **Page/line No.** |
| 18. | Line 76: One would assume the Maasai pastoral systems are well documented. | We went through various literary works and found that the existing documentation of the Maasai pastoral systems is lacking the well characterized Maasai Alalili systems as indigenous/traditional rangeland management strategy. | Page #6  Line 91 |
| 19. | Lines 186: “All Alalili samples were visited…” do you mean villages with Alalili rangelands? | We visited the sampled Alalili systems that are located in the mapped villages with the proposed land use categories | Page #12  Line 231 |
|  | | | |
| **Discussion** | | | |
| **S/N** | **Comments** | **Response** | **Page/line No.** |
| 20. | Line 426: The study described/categorise not obtained. | The statement has been included in section 4.1 | Page #27  Line 523 |
