# Supplementary material for: Sustaining indigenous Maasai Alalili silvo-pastoral conservation systems for improved community livelihood and biodiversity conservation in East African rangelands
Source: PLoS One. 2024 May 16;19(5):e0303649. doi: 10.1371/journal.pone.0303649 (PMC11098305; doi:10.1371/journal.pone.0303649)
Supplement: Attachment — Submitted filename: Response to Reviewers.docx [file pone.0303649.s004.docx]

The Nelson Mandela African Institution of Science and Technology,

P.O.BOX 447,

Arusha-Tanzania.

E-mail: [elkana.hezron@nm-aist.ac.tz](mailto:elkana.hezron@nm-aist.ac.tz)

Mobile No: +255 766 014 232

21^st^ April, 2024

Dr. Emily Chenette (Editor – in – Chief),

PLOS ONE - Public Library of Science.

Dear Sir/Madam,

**RE: Manuscript titled: Sustaining indigenous *Maasai* *Alalili* silvo-pastoral conservation systems for improved community livelihood and biodiversity conservation in East African rangelands**

Refer to the subject above.

Following the minor revision of our aforementioned article, hereby we would like to re-submit the manuscript for publication consideration in PLOS ONE.

This article is re-submitted to PLOS ONE after addressing the minor comments raised by the editor and reviewers (Table below). All authors have agreed to the re-submission of the manuscript to your journal, and have no conflict of interest. Two citations have been added to the reference list citing the software and tools used for data analyses. Also, one conference paper and one research article have been cited in the text to describe the IUCN categorization standards of land use and tenure types liaised with the Tanzanian land use and tenure policies. The citations have been added to the reference list.

We will be delighted if you consider our manuscript acceptable for publication in PLOS ONE.

Sincerely on behalf of the authors

Elkana Hezron

**EDITOR’s COMMENTS ADDRESSED**

| **General comment** | | **Response** |
| --- | --- | --- |
| Thank you for submitting your manuscript to PLOS ONE. After careful consideration, we feel that it has merit but does not fully meet PLOS ONE’s publication criteria as it currently stands. Therefore, we invite you to submit a revised version of the manuscript that addresses the points raised during the review process. | | Thank you for the compliment, we have addressed all the comments that were raised by reviewers as indicated in this table and the revised manuscript. |
|  | | |
| **Other specific comments** | | |
| **S/N** | **Comments** | **Response** |
| 1. | Please make sure to address the final concerns of reviewer #1. | We have addressed the final concerns raised by reviewer #1 as shown in the next table below. |
| 2. | Please review your reference list to ensure that it is complete and correct. If you have cited papers that have been retracted, please include the rationale for doing so in the manuscript text, or remove these references and replace them with relevant current references. Any changes to the reference list should be mentioned in the rebuttal letter that accompanies your revised manuscript. If you need to cite a retracted article, indicate the article’s retracted status in the References list and also include a citation and full reference for the retraction notice. | Thank you for the observation, the reference list has been reviewed for perfection. |

**REVIEWER #1: COMMENTS ADDRESSED**

| **S/N** | **Comments** | **Response** | **Page/line No.** |
| --- | --- | --- | --- |
| 1. | For Table 3 and 4 include precise p-values for all values greater than or equal to 0.001, in line with PLOS ONE Submission guidelines. For smaller values “P < 0.0001” is neater. | The p-values in Tables 3 and 4 have been corrected to suit PLOS ONE guidelines as suggested. | Page #24 – 25  Table 3  Page #26  Table 4 |
| 2. | A careful reading of the manuscript is necessary to ensure that all in-text citations are properly referenced, including the tools and software used. | Software and tools used for data analyses are also cited accordingly in the text and listed in the reference section.  Also, all references in the list have been reviewed and appropriately cited in the text. | Page #11  Line 201  Page #15  Line 290 |
| 3. | Regarding the GEE model, the correlation structure is specified as "independent," suggesting no correlation between responses within subjects. Is this assumption accurate? Given the potential correlation among explanatory variables, the correlation structure "exchangeable" may be more suitable. | We are grateful for this observation. Both "independent" and "exchangeable" assumptions were tested, and only a very little variation in the data was observed. However, we have opted to pick the "exchangeable" assumption for suitability as suggested. | Page #15  Lines 292, 294  Page #24 – 25  Table 3  Page #26  Table 4 |
