# Supplementary material for: Sustaining indigenous Maasai Alalili silvo-pastoral conservation systems for improved community livelihood and biodiversity conservation in East African rangelands
Source: PLoS One. 2024 May 16;19(5):e0303649. doi: 10.1371/journal.pone.0303649 (PMC11098305; doi:10.1371/journal.pone.0303649)
Supplement: Attachment — Submitted filename: Reviewer comments.docx [file pone.0303649.s002.docx]

Reviewer’s comments

Sustaining indigenous Maasai Alalili silvo-pastoral conservation systems for improved community livelihood and biodiversity conservation in East African rangelands

The manuscript describes the indigenous Maasai Alalili silvo-pastoral conservation systems of livestock keeping, which can best be described as a traditional, rotational grazing patterns or a modern fodder bank systems where a large portion of land is reserved for dry spells or drought. These traditional systems have been successful in the past but current socioeconomic and political factors threatened their continued existence. Hence, there is a need to understand how these systems are currently categorised across livestock keeping communities in northern Tanzania, and how best they can be sustained. I have provided here a few suggestions on how the manuscript can be improved.

Overall major comments

Although the indigenous Maasai Alalili silvo-pastoral conservation systems have been successful in the past, it is no longer sustainable given the current human population growth, allocation of lands to conservation, climatic and socio-political tendencies in northern Tanzania. In addition, droughts have decimated rangelands, and many pastoral communities in Ngorongoro district are being relocated to other regions of the country as part of the government land use transformation plans. Despite the importance of the Maasai Alalili silvo-pastoral conservation systems, current government policies would suggest that the practice might phase out in the next few years. The impacts are already obvious. Hence, I would expect the authors to integrate these issues in relevant parts of the manuscript. For example, how would these current policies affect the Maasai Alalili silvo-pastoral conservation systems?

In the data collection process, insufficient information was provided on how the mapping process was conducted and how the authors could have determined the sizes of each rangeland/**Alalili**. These details are important to allow reproducibility of the results that determine the sizes of each *Alalili,* which are key results of the manuscript.

Although using Google satellite images for northern Tanzania might be a reasonable verification process, these are time sensitive information, and the image dimensions and size of rangeland will depend on the season they were captured. In addition, Google satellite images for low resource settings are usually several years old.

The methods for classification of communal and private Alalili should be integrated as part of the study design. I would expect such important data to be collected through a standard process with detail methodology on how the participatory process was conducted, number of participants per village and category of individuals etc. Similar details would be required for the semi-structured interviews. Appending a statement at the end of the paragraph that participatory field visits and semi-structured interviews were used is not very convincing because these are methods that require substantial details.

The discussion section was also not clear and difficult to follow: on one hand the authors were making a case for more communal Alalili systems given that they are better managed; and on the other hand there were suggestions that efforts should be directed towards increasing the number and size of private Alalili.

Specific major comments:

Abstract

A brief definition of the Alalili systems in the context of other systems should have been described in the beginning of the abstract.

Line 18: What proportion of the communal systems have vanished? How many are left? Adding more quantitative and statistical information when findings are presented is important for the reader to understand the evidence.

Introduction

Line 68: In Tanzania what proportion of livestock are kept through Ngitili, Milanga is Radanenda and Alalili?

Does all Maasai population practice Alalili? If not, what are the other major types of surviving strategies the Maasai use?

Methods

Line 90: What districts are the Maasai pastoral society located in in Northern Tanzania?

Line 162-163: What are the justifications for the sampling methods used, e.g. why was a combination of purposive and/or purposive stratified sampling techniques used in the study?

Line 167: These details fit better in the study area description.

Line 175- 179: How many key informant interviews were conducted per district to identify villages that practice Alalili systems? A village was the sampling unit? Where in the districts were these interviews conducted? Was the interview conducted just once for each district? This must be very clear because it an important aspect of the methods, given that the interviews provided valuable information that informed the list of all villages that practice Alalili systems? If so, how was members of the VEO/WEO, and members of village rangeland management committee selected? These individuals are unique for each village in the districts. For example, Longido has more than 51 villages and each village has a VEO.

Line 179-180: Again, please clarify “A total of 298 Alalili systems…”. Are these the number of villages (that is the sampling unit) that practice Alalili systems out of all (n=?) the villages in study districts? What systems does these other villages or communities or clans practice? Why 40%, what are the justification for the sample size of 119?

Line 182: How was the Empirical evidence-based method (Ground-truthing technique) performed?

Lines 185-196: More detail is required throughout this section. Every single village sampled was visited and mapped to verify the exact land use types? So, how was the ground-truthing data collected in each village? For example, as indicated in S1 table, a single waypoint/coordinates for each Alalili system/rangeland? What location was the waypoint collected, centre or edge of the Alalili? Four radii were recorded from the centre to the north, south, east and west directions assuming that Alalili has irregular polygon- how was this recorded (e.g. using transect), and with what instruments (e.g. handheld GPS and paper maps)? And where are the results, given that the local field assistant for each specific village only recorded the average radius, and only a single waypoint was recorded in the S1 table? These details are important to allow reproducibility of the results that determine the sizes of each Alalili, which are important results for the manuscript. Line 191: How was the verification performed? What was the year and date the Google satellite image in QGIS environment was captured? How was the digitisation process performed?

Lines 195: What are participatory visits? How were the semi-structured interviews conducted? Where is the questionnaire?

Results

Line 222: Again, it is difficult to understand this classification when the method description was not adequate.

Figure 5: Are there no other types of land uses in the study area?

Figures 6 and 7: What information was used for this map classification? Ngorongoro district has NCA, Loliondo Game Controlled Area and village lands with significant human settlements around Loliondo town and Wasso based on 2012 census. Has this changed? Longido also has village lands around Longido town and Namanga wards.

Discussion

Line 426 - 438: This discussion is not very clear because Maasai rangelands in northern Tanzania are largely communal with a small proportion being private, which is expected given that Maasai communities in Tanzania have not partition communal lands as it is currently being done in Kenya. Therefore, the result does not suggest that household level practice is being threatened. In fact, it is the opposite as more communal lands (18%) are being acquired by individual for raising livestock and other personal use. The result present does not show if this situation has changed.

Line 428: Again, the results do not show any changes, it only categorises rangeland across land use types.

Minor comments:

Introduction

Line 76: One would assume the Maasai pastoral systems are well documented.

Lines 186: “All Alalili samples were visited…” do you mean villages with Alalili rangelands?

Discussion

Line 426: The study described/categorise not obtained.
